# Neural responses for evaluating self and mother traits in adolescence depend on mother–adolescent relationships

**DOI:** 10.1093/scan/nsz023

**Published:** 2019-04-04

**Authors:** Renske Van der Cruijsen, Renate Buisman, Kayla Green, Sabine Peters, Eveline A Crone

**Affiliations:** 1 Department of Developmental Psychology, Leiden University, Leiden AK, the Netherlands; 2 Institute for Brain and Cognition, Leiden University, Leiden AK, the Netherlands; 3 Forensic Family Science and Youth Care Studies, Leiden University, Leiden AK, the Netherlands

**Keywords:** adolescence, mother–adolescent relationship, family interactions, fMRI, medial prefrontal cortex

## Abstract

An important task in adolescence is to achieve autonomy while preserving a positive relationship with parents. Previous fMRI studies showed largely overlapping activation in medial prefrontal cortex (mPFC) for evaluating self and close-other traits but separable activation for self and non-close other. Possibly, more similar mPFC activation reflects closeness or warmth in relationships. We investigated neural indicators of the mother–adolescent relationship in adolescents between 11 and 21 years (*N* = 143). Mother–adolescent relationship was measured using (i) mothers’ and adolescents’ trait evaluations about each other, (ii) observations of warmth, negativity and emotional support in mother–adolescent conversation and (iii) similarity in adolescents’ neural activation for evaluating self *vs* mother traits. Results showed relatively more similar mPFC activation in adolescents who evaluated their mothers’ traits more positively, suggesting that this is possibly a neural indicator of mother–adolescent relationship quality. Furthermore, mid-adolescence was characterized by more negative mother–adolescent interaction compared to early and late adolescence. This effect co-occurred with mid-adolescent peaks in dorsal striatum, dorsal anterior cingulate cortex and superior temporal sulcus activation in evaluating traits of self *vs* mother. These results suggest more negative relationships and stronger self-focus in mid-adolescence.

## Introduction

A critical task during adolescence is to achieve autonomy while preserving a positive relationship with parents (Allen *et al.*, 1994). Maintaining a positive relationship with parents is important, as this promotes better well-being and less delinquency in adolescents (Hair *et al.*, 2008). Adolescents develop a more positive self-concept when they experience supportive parenting, emotional availability of parents and when mothers express competence beliefs about their adolescents (Dekovíc and Meeus, 1997; Gniewosz *et al.*, 2015; Lazarides *et al.*, 2015; Babore *et al.*, 2016). In contrast, negative family factors, such as household chaos, can negatively influence social competence development, and more household chaos is associated with reduced neural signals for cognitive control (Kim-Spoon *et al.*, 2017).

During adolescence, there is a transition in parent–child relationships such that there is an increase in arguing frequency between parents and teenagers (Steinberg and Morris, 2001). This increase in mild conflict is accompanied by a decline in feelings of closeness between parents and adolescents (Steinberg and Morris, 2001; McGue *et al.*, 2006; Smetana *et al.*, 2006). More specifically, both parents and adolescents report less frequent expressions of positive emotions and more frequent expressions of negative emotions compared to pre-adolescent children (Larson *et al.*, 1996; Steinberg and Silk, 2002). Adolescents’ affect towards their family shows a temporary decline in mid-adolescence (Larson *et al.*, 1996; Laursen *et al.*, 1998). Similarly, perceived affect and warmth from family members decrease in early adolescence and increase again in late adolescence (Larson *et al.*, 1996; Shanahan *et al.*, 2007). Some adolescents experience more negative parent–adolescent relationships than others, but it is currently not well understood how this affects closeness between adolescents and parents.

A novel direction in understanding the parent–adolescent relationship is by using insights from cognitive and social neuroscience. Prior studies already demonstrated bidirectional relationships between parents and children in terms of physiological responses in an interaction context. For example, cortisol levels of fathers, mothers and adolescents collected before and after a conflict discussion task were positively related to one another, such that fathers’ cortisol predicted later adolescents’ cortisol, which in turn predicted later mothers’ cortisol levels (Saxbe *et al.*, 2014a). Additionally, insights from brain imaging studies are informative for understanding individual differences in the relationship between parents and adolescents. A prior study showed that the relationship between parent aggression and subsequent adolescent aggression was mediated by brain activation in response to parent-related stimuli in youth between 15 and 17 years, in regions associated with salience and socioemotional processing such as the insula, right amygdala, thalamus and putamen (Saxbe *et al.*, 2016). Finally, a recent study in adults showed that self-referential processing in the medial prefrontal cortex (mPFC) was sensitive to individual differences in caregiving experiences (Noll *et al.*, 2018), but no prior studies have examined how self-referential processing and parenting are related in adolescence. Therefore, the current study aimed to gain a better understanding of the behavioral and neural correlates of the mother–adolescent relationship by combining (i) mothers’ and adolescents’ self-reported evaluations of each other, (ii) real-life observations of mother–adolescent interactions and (iii) fMRI-measurements of evaluating self *vs* mother traits.

### Neural activation for self, close and distant others

Prior studies in adults showed consistent overlapping activation in the mPFC for evaluating traits of the self (Denny *et al.*, 2012) and close others, relative to more distant or public others (Murray *et al.*, 2012). The level of similarity within mPFC has been related to the level of closeness or familiarity (Van Overwalle, 2009; Krienen *et al.*, 2010; Raposo *et al.*, 2011), the level of warmth and competence (Harris and Fiske, 2007), or, when close others were participants’ mothers, the degree of attachment to the mother (Ray *et al.*, 2009). Directly relating indicators of the mother–adolescent relationship to neural activation for evaluating self *vs* mother can provide important new insights in the understanding of individual differences in mother–adolescent relationships.

To date, only two studies investigated self- *vs* close-other processing in children and young adolescents. The first study (participants 7–13 years) showed that children remember words encoded with reference to their mother better than with reference to themselves, whereas this effect was reversed in young adolescents (Ray *et al.*, 2009). The difference in self *vs* mother memory was related to more ventral anterior cingulate cortex (ACC)/mPFC activation for self *vs* mother. Therefore, this study suggests a separation of self from mother during early adolescence (Ray *et al.*, 2009). The second study showed similar mPFC activation for evaluating traits of self and one’s best friend in both young adolescents (age 11–14) and young adults (age 22–31; Jankowski *et al.*, 2014).

As neural similarity or differentiation may provide important insights into the relative strength of the mother–adolescent relationship, this study investigates whether individual differences in mother–adolescent relationship quality are associated with increased similarity for evaluating self and mother in mPFC (Ray *et al.*, 2009; Noll *et al.*, 2018). In addition, we performed exploratory analyses for associations with other regions that have been reported in prior studies in relation with self-concept development, including the ventral striatum and the temporoparietal junction (TPJ; Pfeifer *et al.*, 2009; Pfeifer and Peake, 2012; Jankowski *et al.*, 2014). An additional goal was to assess developmental changes in mother–adolescent relationships. To test these questions, participants (*N* = 143; 11–21 years) evaluated their own and their mother’s traits in an fMRI paradigm, and mothers evaluated adolescents’ traits via an online questionnaire. Additionally, we assessed observational measures for warmth, negativity and emotional support in real-life mother–adolescent discussions about conflictual topics (Buisman *et al.*, in press). Neural indicators of the mother–adolescent relationship were studied by examining similarity and differentiation in adolescents’ neural activation for evaluating self *vs* mother traits.

First, we expected that positivity of evaluations about the mother (for the adolescent) and the adolescent (for the mother) would be positively correlated to warmth and emotional support in mother–adolescent interactions, as these measures are hypothesized to reflect the mother–adolescent relationship. Second, we expected overlapping brain activation for adolescents evaluating themselves and their mothers in mPFC (Zhu *et al.*, 2007; Vanderwal *et al.*, 2008; Murray *et al.*, 2012; van der Cruijsen *et al.*, 2017), the precuneus and temporal–parietal junction (van der Cruijsen *et al.*, 2017). A prior study also demonstrated an important role for the striatum when reflecting on social traits from the perspective of peers in adolescents (Jankowski *et al.*, 2014), but it is not yet known if the striatum is also involved in evaluating self *vs* mother traits. Third, we tested whether increased similarity in neural activity for evaluating self *vs* mother traits was associated with higher mother–adolescent relationship quality (Denny *et al.*, 2012; Murray *et al.*, 2012). To this end, we hypothesized that mPFC activity for evaluating self *vs* mother traits was related to adolescents’ and mothers’ positivity about each other’s traits and to warmth and emotional support in mother–adolescent interactions.

Regarding possible developmental changes, we expected that mid-adolescents are more negative in interaction with their mothers compared to early and late adolescents (Larson *et al.*, 1996; Laursen *et al.*, 1998). A similar pattern was expected for mothers in interaction with their children (Larson *et al.*, 1996). We explored whether differences in neural activation for evaluating self *vs* mother would show a comparable age-related change as the pattern observed for mother–adolescent interactions. Therefore, we tested for quadratic patterns of neural sensitivity for self–mother trait evaluations (see also Braams *et al.*, 2015).

## Methods

### Participants

Healthy adolescents (*N* = 160; age 11–21) participated in the Leiden Self-Concept study. Part of the fMRI self-evaluation data have previously been reported (van der Cruijsen *et al.*, 2018). All participants were right-handed and reported normal or corrected-to-normal vision. Parents (about their child, in a phone conversation prior to inclusion) and participants (in an online questionnaire) reported no psychiatric or neurological diagnoses. We excluded 17 participants for the following reasons: no complete data for self and mother evaluations (*n* = 2); participant’s mother did not complete the trait evaluations about her child (*n* = 6); fMRI-related exclusions (*n* = 11), specifically excessive head movements during the fMRI scans (>3 mm across the full run; *n* = 8), not completing all scans (*n* = 2) and technical error (*n* = 1). The resulting sample consisted of 143 adolescents [mean age (*M*_age_), 16.17 years; s.d., 2.9; 79 females]. In all analyses including measures of mother–adolescent interaction, the sample size was reduced to *n* = 93 (*M*_age_, 15.29; s.d., 2.8; 52 females), as 50 participants did not engage in the mother–adolescent interaction task because the mother was not available to attend the session.

All participants and both parents of minors signed informed consent. The University Medical Ethical Committee approved the study. Participants were pre-screened for MRI contra-indications and usage of psychotropic medication. A radiologist viewed all scans and no clinically relevant findings were observed.

**Fig. 1:**
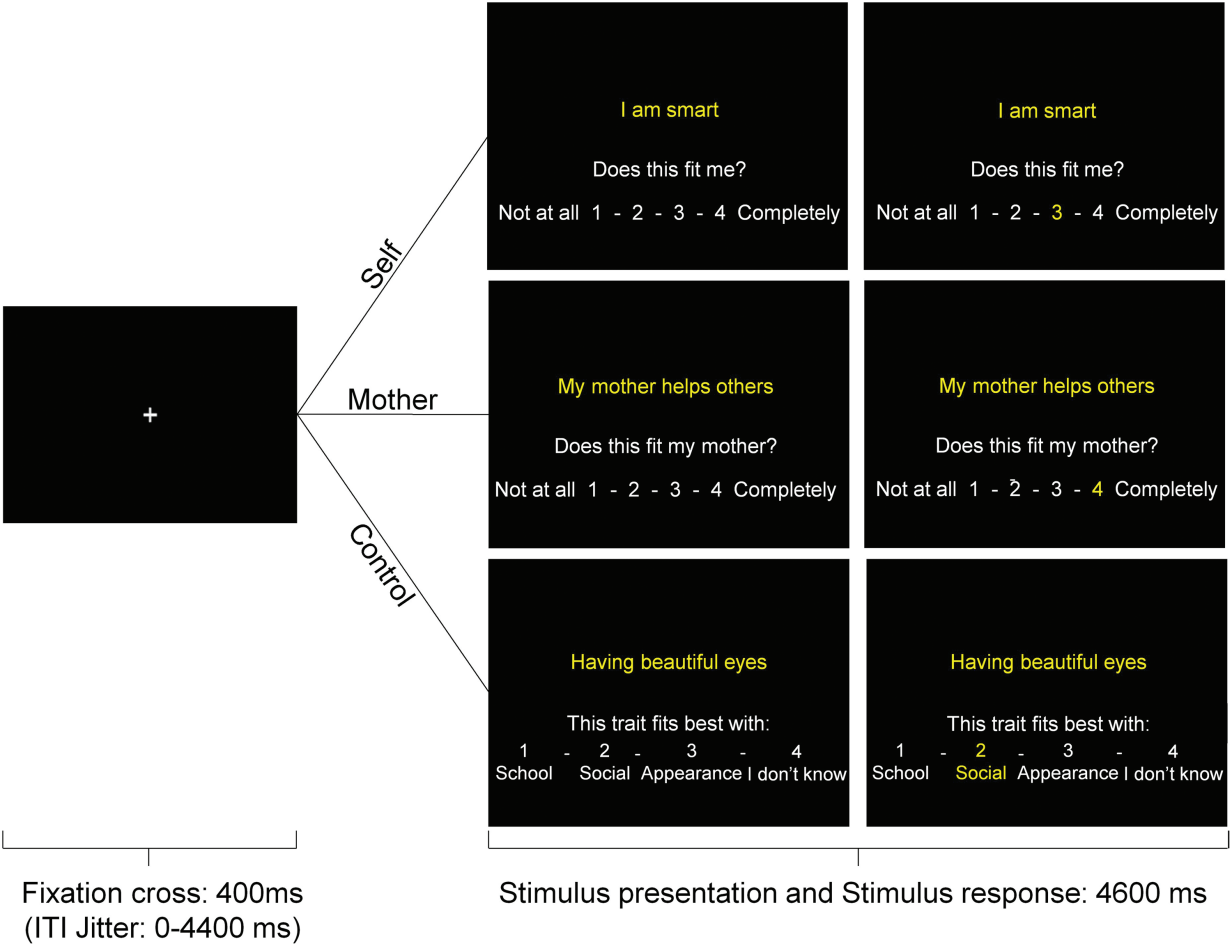
Example of a trial in the self, mother and control conditions.

**Table 1:** Correlations between mother–adolescent interaction and trait evaluations of adolescence and mother

			**Evaluations of**		**Adolescent**		**Mother**		
			**Adolescent about mother**	**Mother about adolescent**	**Warmth**	**Negativity**	**Warmth**	**Negativity**	**Emotional support**
A. *Evaluations of:*									
Adolescent about self		*R*	**0.415**	**0.438**	0.198	−0.149	0.169	−0.049	−0.154
		*P*	**0.000**	**0.000**	0.058	0.155	0.106	0.644	0.141
Adolescent about mother		*R*	—	**—**	0.049	−0.082	0.048	−0.055	0.119
		*P*			0.644	0.439	0.649	0.598	0.258
Mother about adolescent		*R*	—	**—**	**0.412**	−0.189	**0.384**	**−0.252**	0.044
		*P*			**0.000**	0.071	**0.000**	**0.015**	0.676
B. *Observations of:*									
Adolescent	Warmth	*R*	—	—	**—**	**−0.523**	**0.527**	**−0.317**	0.139
		*P*				**0.000**	**0.000**	**0.002**	0.185
	Negativity	*R*	—	—	**—**	**—**	**−0.421**	**0.448**	−0.169
		*P*					**0.000**	**0.000**	0.107
Mother	Warmth	*R*	—	—	**—**	**—**	**—**	**−0.596**	**0.340**
		*P*						**0.000**	**0.001**
	Negativity	*R*	—	—	**—**	**—**	**—**	**—**	**−0.366**
		*P*							**0.000**

Note: Bold is equal to significant result.

**Fig. 2:**
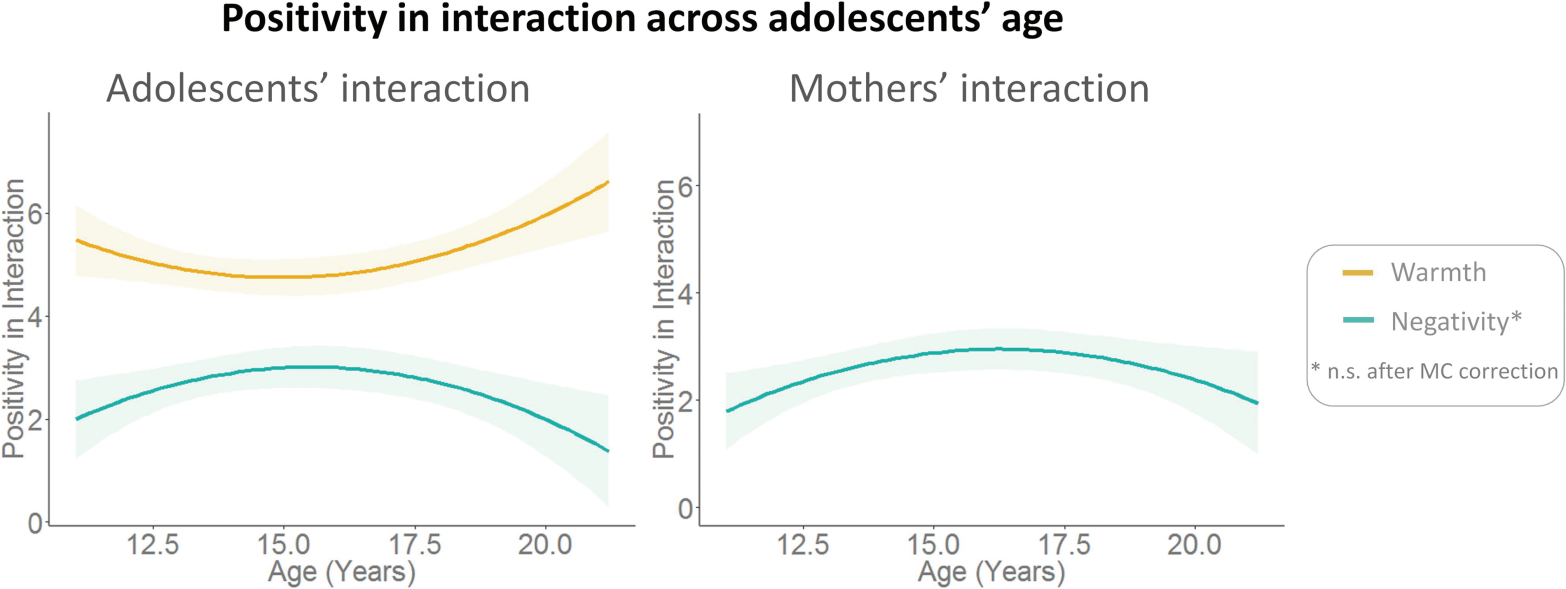
Measures of warmth and negativity in mother–adolescent communication, related to age. Only significant results are shown. Adolescents showed a dip in warmth and a peak in negativity in interaction with their mothers in mid-adolescence. Mothers showed a peak in negativity in interaction with their children when they are in mid-adolescence.

### Task description

#### Mother–adolescent conflict interaction task

To investigate interaction patterns between mothers and adolescents, we used a mother–adolescent conflict interaction task (family interaction task; Allen *et al.*, 2003). This task was previously found to be effective in measuring mother–adolescent relationship behaviors (Eisenberg *et al.*, 2008; Buisman *et al.*, in press). First, mothers and adolescents selected topics that regularly cause conflict from a list. The researcher selected two of these topics, based on which topics were rated highest, preferably by both mother and adolescent. Mothers and adolescents were then asked to discuss these conflictual topics for 10 min, thereby trying to reach a consensus. They were told to start with one topic, and only continue to the second if there was time left after the first topic. Most discussed topics were bedroom cleaning, bedtime, cleaning up clothes and homework. All interactions were videotaped and coded using the Supportive Behavior Task Coding Manual Version 1.1 (Buisman *et al.*, in press).

Behaviors of warmth, negativity and emotional support were rated for mothers and adolescents. Warmth is the extent to which they showed that they care about, value and genuinely like the other. It encompasses verbal expressions (f.e. verbally empathizing), warm facial expressions, a warm tone of voice and body postures or behaviors that indicate warmth and the intention to build the relationship (f.e. touching). Warmth was rated on a scale of 1 (no signs of warmth; ‘You cannot tell if the person likes or cares about the other’) to 9 (clear signs of warmth; ‘The participants’ behavior overall gives a warm feeling to the interaction’). Negativity encompasses expressions of tension, hostility, dissension or antagonism directed at the other, for example loud sighing, interrupting the other or eye rolling. Negativity was rated on a scale of 1 (demonstrations of negativity are absent) to 9 (the person is very negative; ‘The negativity endures throughout the discussion and is disruptive to the interaction’). Negativity scores of mothers were skewed to the right. Therefore, these scores were logarithmically transformed. Emotional support captures the extent to which a person indicates an understanding or supports the feelings of the other, for example by naming the emotion, sympathizing or recognizing the other’s feelings. The 9-point rating scale ranged from 1 (absence of emotional support; ‘No attempts to emotionally support the other are made’) to 9 (high emotional support; ‘The supporter clearly recognizes the other’s emotional distress and makes clear attempts to draw the other out’). There was little variance in the adolescent emotional support ratings, likely because mothers and not adolescents were expected to be supportive during conversations. Therefore, we did not include adolescent emotional support ratings in our analyses.

Mother–adolescent interactions were coded using a coding system adapted from Buisman *et al.* (in press), who also validated the coding system. For the purpose of the current study, Buisman (here second author) trained the third author and a research assistant to reliably code the mother–adolescent interactions using this system. Before coding the interactions of the current study, the second author provided extensive training in the coding system, which the coders practiced using 18 training videos (36 targets). After training, 27 videos from the current study (54 individuals) were coded to assess reliability. Inter-rater reliability between the observers was adequate to good on all measured constructs (warmth: α = 0.78; negativity: α = 0.82; emotional support: α = 0.86). Intraclass correlations (single measures and absolute agreement) were 0.61 for warmth, 0.69 for negativity and 0.75 for emotional support. All other videos were coded (*n* = 66) over the next 3 months. To minimize observer drift, videos were discussed during regular meetings (once every 2 or 3 weeks). Coders were randomly divided across participants and mothers before coding started. For the participants and mothers in videos used for the reliability set, the scores from the beforehand assigned coder were used in the analyses. All other participants and mothers were coded by one person.

#### fMRI task

In the fMRI task, participants were asked to evaluate how well short trait sentences described themselves and their mothers on a scale of 1 (not at all) to 4 (completely) by pressing buttons with their index to little finger of their right hand (Figure 1). In the self-condition, participants viewed 60 sentences describing traits in the physical, academic or prosocial domain, with 20 traits per domain (10 positive and 10 negative). In the mother-evaluation condition, participants evaluated the same sentences, except the academic trait sentences were omitted as these were not applicable to adults (e.g. ‘getting good grades’), resulting in 40 trait sentences in the mother condition. In the baseline control condition, all response demands were the same, but participants sorted 20 trait sentences (10 positive and 10 negative) into four categories: school, social, appearance or I don’t know. Additionally, outside of the scanner, mothers evaluated the same 60 traits (in academic, physical and prosocial domains) about their adolescents. Negative items were reverse scored and averaged with the positive items for adolescent and mother ratings separately. This way, an index of positivity of adolescents about their mothers, and (via an online questionnaire) of mothers about their children was obtained, as indicators of the mother–adolescent relationship.

**Fig. 3:**
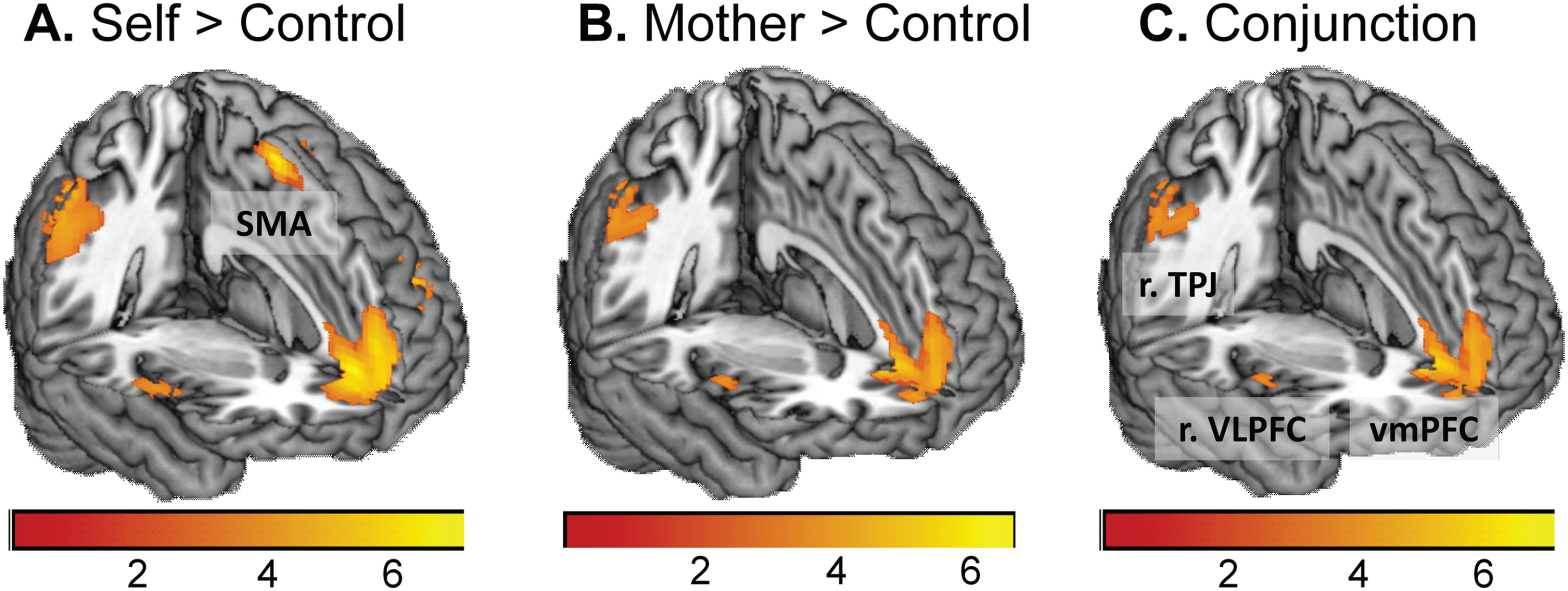
Activation in the contrast self>control, mother>control and the conjunction of these contrasts. Overlapping activation for evaluating self and mother in mPFC, right TPJ and right vlPFC.

**Fig. 4:**
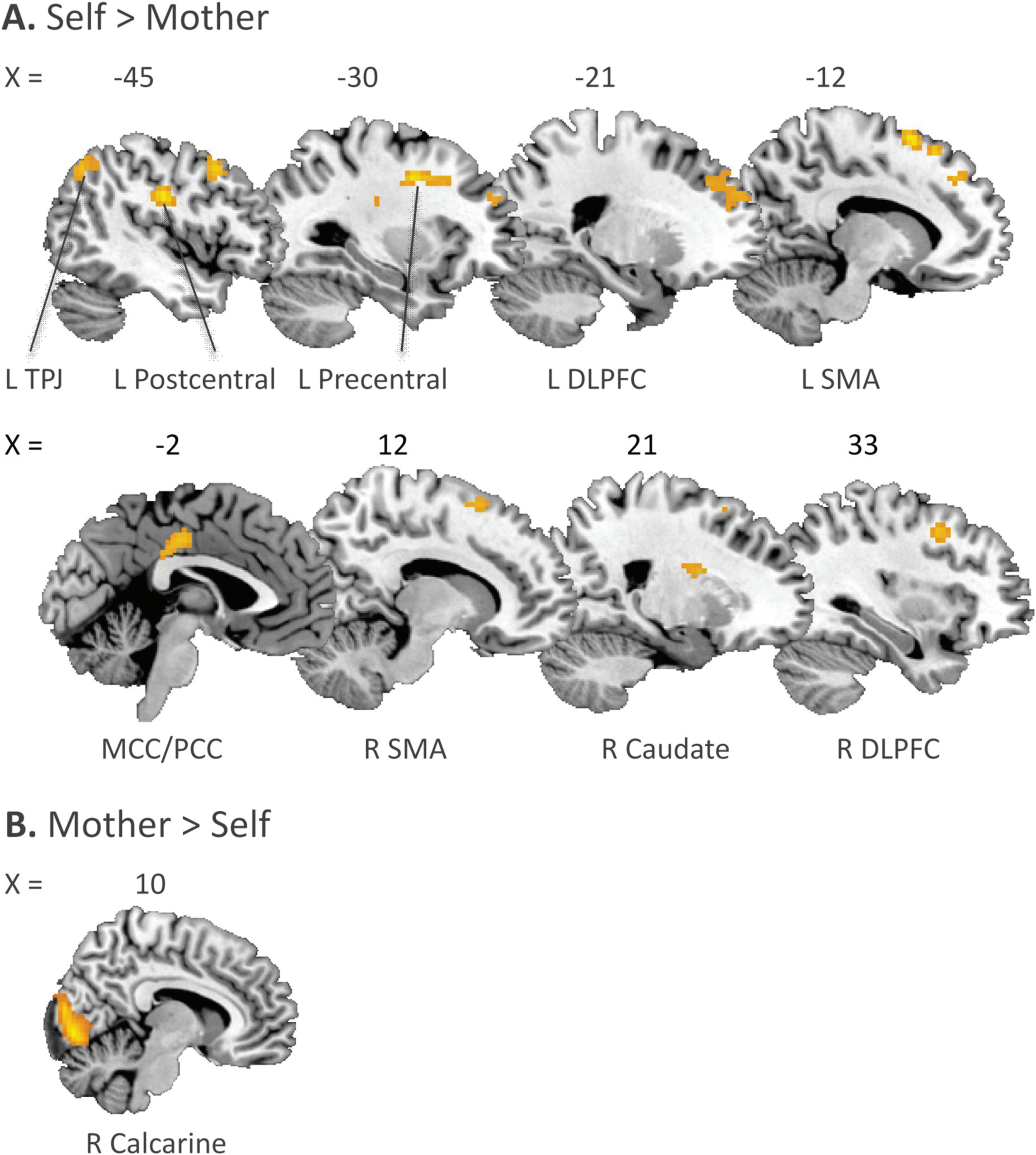
(**A)** Activation in the contrast (self>control)>(mother>control). **(B)** Activation in the contrast (mother>control)>(self>control).

Within the MRI paradigm, the three conditions (self, mother and control) were completed in three separate runs, within which traits were presented in a pseudorandomized order regarding domains. Each trial began with a 400 ms fixation cross, after which the trial displaying a short trait sentence and the question how much this trait was applicable was presented for 4600 ms. When participants responded, the number they chose turned yellow for the remaining stimulus time to assure participants that their choice was registered. If participants failed to respond within the time frame, the phrase ‘Too late’ was displayed for 1000 ms. Trials where participants failed to respond were excluded from the analyses. Too late responses occurred on 1.1% of trials in the self condition, on 1.5% of trials in the mother condition and on 0.6% of trials in the control condition. Optseq was used to optimize the trial order and to add jittered intertrial intervals, varying between 0 and 4.4 s.

### fMRI statistical analyses

For this study, we derived our hypothesis concerning the mPFC from prior literature. Brain–behavior relations in other neural regions, including the ventral striatum, precuneus and TPJ, were tested in an explorative way.

Details on fMRI data acquisition and pre-processing can be found in the supplement. The data were analyzed using SPM8 (Wellcome Department of Cognitive Neurology, London). The fMRI time series were modeled as a series of zero-duration events convolved with the hemodynamic response function. A first-order autoregressive model [AR(1)]-corrected for serial autocorrelations and low-frequency signals were removed using a high-pass filter (120 s). Modeled events of interest for the self condition were ‘Self-Academic-Positive’, ‘Self-Academic-Negative’, ‘Self-Physical-Positive’, ‘Self-Physical-Negative’, ‘Self-Prosocial-Positive’ and ‘Self-Prosocial-Negative’. The same events, except for ‘Academic-Positive’ and ‘Academic-Negative’ were modeled for the mother condition. For the control condition, only one event of interest was modeled: ‘Control’ (collapsed across domains and valences). Trials in which participants failed to respond in time were modeled as events of no interest. Six motion regressors were added to the model, and participants who moved more than 3 mm were excluded from further analyses. The resulting contrast images, computed on a subject-by-subject basis, were submitted to group analyses.

To investigate neural indicators of the mother–adolescent relationship, we performed two whole-brain one sample *t*-tests for the contrasts self>control and mother>control, followed by a conjunction analysis. The self-evaluation trials and the mother-evaluation trials were collapsed across domains and valences and compared to the control trials. The results for self>control have previously been reported (van der Cruijsen *et al.*, 2018), but the current sample differed slightly from the prior sample due to missing values in mother ratings. Therefore, the results for this group are reported again. Next, we used Imcalc toolbox in SPM8 to calculate the contrast for (self–control)>(mother–control).

To test for neural markers of the mother–adolescent relationship, we investigated neural responses during self *vs* mother evaluations and related these to behavioral measures of the mother–adolescent relationship (mothers’ and adolescents’ positivity about each other’s traits and warmth, negativity and emotional support in mother–adolescent interactions). We used these measures as positive and negative regressors in the (self–control)>(mother–control) contrast. To test for developmental changes we used age and age^2^ as a regressor in this contrast. Within these analyses, larger differences for evaluating self *vs* mother reflected relatively less neural similarity, whereas smaller differences for evaluating self *vs* mother reflected relatively more neural similarity in brain activation for evaluating self and mother traits. Age was a covariate of no interest in all regressions except the ones testing linear age effects.

For the behavioral results we used Bonferroni correction for multiple comparisons (MC) adjusting for correlated variables (http://www.quantitativeskills.com/sisa/calculations/bonfer.htm; Sankoh *et al.*, 1997; Perneger, 1998). This resulted in an adjusted significance level (two-sided) of *α* = 0.011 for the eight variables of mother–adolescent relationship (average *r* = 0.26) and *α* = 0.016 for the developmental changes in mother–adolescent interactions (warmth, negativity, emotional support and average correlation *r* = 0.28). We reported when analyses were significant at *P* < 0.05 but did not survive MC correction. For all whole-brain fMRI analyses, we applied False Discovery Rate cluster-level (FDRc) correction (*P* < 0.05) at an initial uncorrected threshold of *P* < 0.001. All uncorrected *t*-value maps can be found on NeuroVault (https://neurovault.org/collections/IQLJYMLX/). These maps also include analyses controled for age and gender. Gender effects were not further explored in this study.

## Results

### Behavioral markers of the mother–adolescent relationship

First, we tested whether behavioral indicators of the mother–adolescent relationship (i.e. ratings of adolescents about their mothers and vice versa and observations of warmth, negativity and emotional support) were related to each other. As can be seen in Table 1, mothers’ ratings of adolescents were positively correlated with observed mother–adolescent interaction.

Second, we investigated the development of mother–adolescent relationships. Curve estimations testing linear and quadratic age patterns showed that positivity of adolescents about their mothers and vice versa did not change with age (all *P* > 0.22). Observed mother–adolescent interactions showed a quadratic age effect for adolescents’ displays of warmth, indicating a dip in mid-adolescence [t(90) = 2.85, *P* = 0.006; *R*^2^ = 0.106, *F*(2,89) = 5.28, *P* = 0.007; Figure 2]. There was also a quadratic age effect on adolescents’ [t(90) = −2.73, *P* = 0.008; *R*^2^ = 0.079, *F*(2,89) = 3.83, *P* = 0.025] and mothers’ [t(90) = −2.35, *P* = 0.021; *R*^2^ = 0.067, *F*(2,89) = 3.22, *P* = 0.044] displays of negativity, with a peak in mid-adolescence. Both developmental patterns of negativity were not significant after MC correction (*P* = 0.016). All other models were not significant (all *P* > 0.14).

### Neural markers of the mother–adolescent relationship

To detect brain regions involved in self evaluations and mother evaluations, we first conducted two whole-brain one-sample *t*-tests for self>control and mother>control. The contrast self>control revealed activation in mPFC, right supramarginal gyrus (TPJ), right ventrolateral prefrontal cortex (vlPFC), left dorsolateral prefrontal cortex (dlPFC), left insula, left superior temporal gyrus (STG) and left supplementary motor area (SMA; Figure 3A; Supplementary Table S1A). The contrast mother>control resulted in similar patterns of activation in mPFC, TPJ and right vlPFC (Figure 3B; Supplementary Table S1B). A conjunction analysis for self>control and mother>control showed overlapping activation in mPFC, right TPJ and right vlPFC (Figure 3C).

**Fig. 5:**
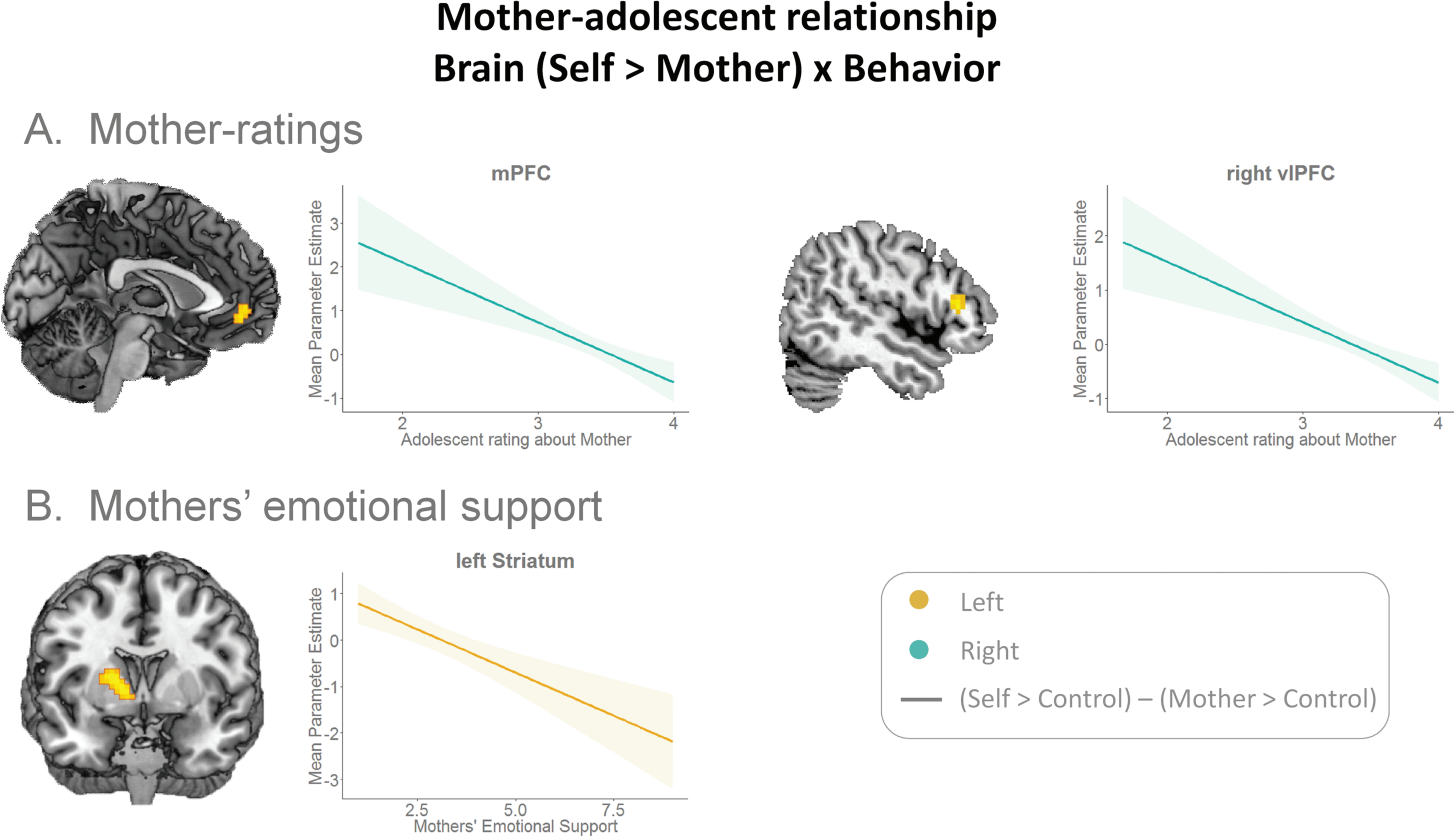
Whole-brain multiple regressions on the self>mother contrast with behavioral measures of mother–adolescent relationship as regressors. **(A)** Stronger mPFC and vlPFC activation for self compared to mother in adolescents who are more negative about their mothers. Similar mPFC activation for self and mother and stronger vlPFC activation for mother than self in adolescents who are more positive about their mothers. **(B)** Relatively stronger left putamen (striatum) activation for mother in adolescents whose mothers showed more emotional support.

**Fig. 6:**
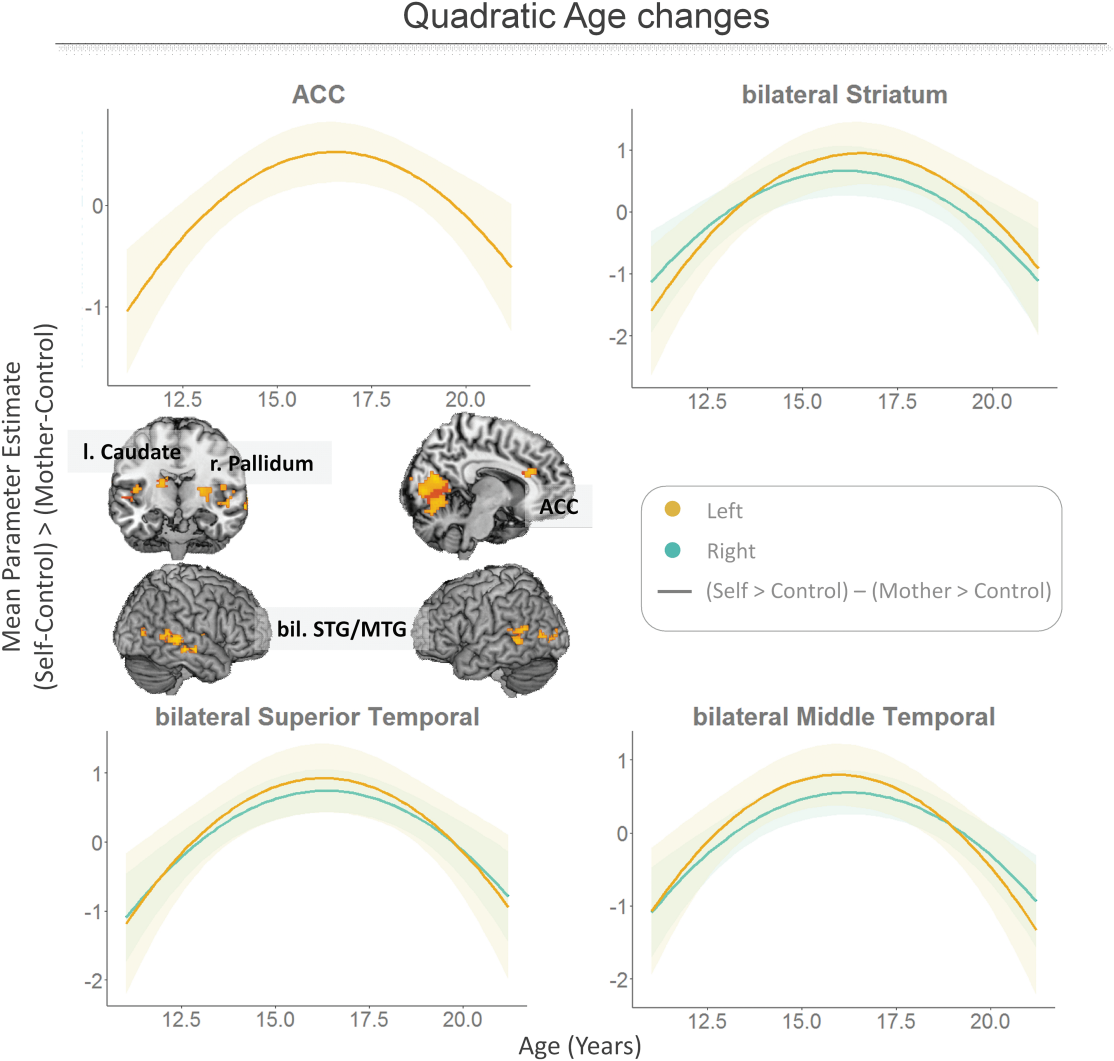
Whole-brain multiple regressions on the (self–control)>(mother–control) contrast with quadratic age as a regressor show relatively stronger activation in ACC, bilateral striatum (right pallidum and left caudate) and bilateral superior and middle temporal gyrus for self *vs* mother in mid-adolescence compared to early and late adolescence.

To investigate differential activation for evaluating self and mother, we constructed the contrasts (self–control)>(mother–control) (self>mother for short) and (mother–control)>(self–control) (mother>self for short). Self>mother resulted in medial/posterior cingulate cortex, bilateral dlPFC, bilateral SMA, left angular gyrus (TPJ), right caudate nucleus (ventral striatum) and left occipital lobe activation (Figure 4A; Supplementary Table S2A). Mother>self resulted in activation in right calcarine gyrus (Figure 4B; Supplementary Table S2B).

#### Relations with trait evaluations and interaction behaviors

To test for neural markers reflecting the mother–adolescent relationship, we investigated whether neural differences for evaluating self and mother were related to behavioral measures of the mother–adolescent relationship. We performed whole-brain analyses on the contrast self>mother, including measures of mother–adolescent relationship as positive and negative regressors [trait ratings and mother–adolescent interaction (emotional support, warmth and negativity)].

Results showed a negative relationship between mPFC and right vlPFC activation and adolescents’ evaluations of mothers, with relatively less mPFC and right vlPFC activity for mother *vs* self in adolescents who evaluated their mothers’ traits more negatively, and more similar mPFC and right vlPFC activation for self and mother in adolescents who evaluated their mothers’ traits more positively (minimum cluster-size FDRc-corrected = 41; Figure 5A).

Next, we performed analyses with observed mother–adolescent interaction measures as regressors. There was a negative relationship between left putamen activation for self>mother and mothers’ emotional support, such that there was relatively more left putamen (striatum) for mother in adolescents who receive more emotional support compared to adolescents who receive less emotional support (minimum cluster-size FDRc-corrected = 54; Figure 5B). For all t-maps and additional t-maps for all above analyses corrected for gender, see https://neurovault.org/collections/IQLJYMLX/. Last, adolescents whose mothers were more negative in their communication, showed relatively more bilateral middle occipital lobe activation for evaluating self *vs* mother (see Supplementary data for more detailed results for bilateral middle occipital lobes).

#### Age differences in self *vs* mother ratings

To investigate developmental changes in activation for self and mother, we performed whole-brain analyses on the self>mother contrast, including linear age or age^2^ as positive and negative regressors. Results did not show significant changes with linear age. However, there was a quadratic mid-adolescent peak in ACC, bilateral dorsal striatum (left caudate nucleus and right pallidum), bilateral superior STG and a related region in Rolandic operculum, bilateral middle temporal gyrus (MTG), left calcarine gyrus and left middle occipital lobe (minimum cluster-size FDRc-corrected = 35). These regions were engaged relatively more for evaluating self *vs* mother in mid-adolescents, compared to early and late adolescents (Figure 6). For all t-maps and additional t-maps for the above analyses corrected for gender, see https://neurovault.org/collections/IQLJYMLX/.

## Discussion

We investigated behavioral measures and neural markers of mother–adolescent relationship quality and the development of this relationship across adolescence, using self-report, observation and fMRI measures. This study showed that differences in neural activation for evaluating self *vs* mother varied as a function of behavioral measures of mother–adolescent relationship. Specifically, activity for self and mother was more similar in mPFC and right vlPFC for adolescents who were more positive about their mothers. Furthermore, activity was relatively stronger for mother than self in the striatum for adolescents whose mothers showed more emotional support in observed interactions.

A second goal was to examine whether there was an adolescent-specific decrease in adolescent–mother relationships. Developmental analyses showed that adolescents, and to a lesser extent mothers as well, were more negative in interaction with the other in mid-adolescence. The same developmental analyses for neural activity revealed that striatal regions and dorsal ACC showed more activity for self *vs* mother in mid-adolescence compared to early and late adolescence. Together these results suggest relatively negative relationships and stronger self-focus in mid-adolescence. These individual differences and developmental differences are discussed in more detail below.

### Individual differences in the mother–adolescent relationship

In prior research, it has been suggested that mother–adolescent relationships influence how adolescents view themselves and others (Dekovíc and Meeus, 1997; Gniewosz *et al.*, 2015; Lazarides *et al.*, 2015; Babore *et al.*, 2016), but the underlying mechanisms are not yet well understood. We took the approach of studying neural responses when rating traits of self and mothers. First, we found that mother–adolescent interactions were indeed related to subjective evaluations of the other’s traits. That is, mothers who showed and received more warmth in observed interactions were more positive about their children’s traits, and mothers who showed more negativity in interaction were less positive about their children. Additionally, mothers’ behavioral indicators of warmth were related to more warmth and less negativity in the adolescents’ behaviors and vice versa. This is in line with previous studies indicating that parent and adolescent behaviors reinforce each other (Saxbe *et al.*, 2014b, 2016).

On the neural level, more similar activation in the regions implicated in evaluating self and close others are thought to reflect closeness or trait warmth (Harris and Fiske, 2007; Krienen *et al.*, 2010; Raposo *et al.*, 2011). Therefore, we examined whether mother–adolescent relationship quality was also related to neural responses in mPFC to evaluating traits of self and mother. Consistent with our hypothesis, this study revealed that mPFC activation for self *vs* mother was related to adolescents’ positivity about their mother (mother positivity), with less differentiation between self and mother in adolescents who reported higher mother positivity. The mPFC has previously been associated with increased self-relevance (Moran *et al.*, 2006; D’Argembeau, 2013; van der Cruijsen *et al.*, 2018), and previous studies showed more neural similarity in mPFC for self and close others, than for self and non-close others (Murray *et al.*, 2012). Therefore, evaluating mothers’ traits is possibly more similar to self for adolescents who are positive about their mothers and might reflect more closeness in the mother–adolescent relationship (Harris and Fiske, 2007; Van Overwalle, 2009; Krienen *et al.*, 2010; Ray *et al.*, 2010; Raposo *et al.*, 2011).

Additionally, adolescents who received more emotional support from their mothers in observed interactions engaged left striatum (putamen) relatively stronger for mother, compared to adolescents who received less emotional support. Previous studies showed involvement of left putamen not only in monetary and social rewards (Izuma *et al.*, 2008; Braams *et al.*, 2015; Wake and Izuma, 2017) but also in processing self-relevance (Enzi *et al.*, 2009; de Greck *et al.*, 2010). This suggests that for adolescents who received more emotional support, thinking about their mothers might elicit a stronger feeling of reward or self-relevance compared to adolescents with less supportive mothers. Future studies are needed to confirm this, and longitudinal designs could reveal whether developmental differences in mother–adolescent relationship quality relate to striatum activity over longer time periods.

In sum, this study tested and confirmed the previously used interpretation that more similar activation in mPFC reflects closeness or warmth in the relationship between the self and a close other (Harris and Fiske, 2007; Krienen *et al.*, 2010; Raposo *et al.*, 2011). Furthermore, this study extends previous findings showing that in addition to parent- and adolescent self-reported behavior (Saxbe *et al.*, 2016), actual observed interactions are related to neural responses in mPFC as well. By showing that self-reported and observed behavioral indicators of the mother–adolescent relationship are associated with adolescents’ brain activation for self and mother, this study provides a first direction in understanding the neural basis of individual differences in mother–adolescent relationships. A next step would be to longitudinally investigate the relationship quality between mothers and adolescents over time and the inter-relations with neural and behavioral self-concept development.

### Development of the mother–adolescent relationship across adolescence

Our second main goal was to investigate the ‘development’ of mother–adolescent relationships. Behaviorally, mother–adolescent interactions decreased in positivity in mid-adolescence, with mid-adolescents displaying less warmth and more negativity compared to early and late adolescents. However, mid-adolescents’ trait evaluations of their mothers do not differ from those of early and late adolescents. Potentially, a strong self-focus (i.e. egocentrism) in mid-adolescence (Harter, 2012) leads these adolescents to communicate less considerately with their parents (i.e. less warm and more negative). Additionally, mothers of mid-adolescents showed more negativity compared to mothers of early and late adolescents. These findings are consistent with previous studies, which reported decreased feelings of closeness and more negative affect in mid-adolescence mainly based on self-reports (Larson *et al.*, 1996; Laursen *et al.*, 1998; Steinberg and Silk, 2002).

To further test whether there was a mid-adolescent focus on self, we examined the neural responses when evaluating traits of self and mothers. Neuroimaging results showed that activation for self *vs* mother in bilateral striatum (right pallidum and left caudate), ACC and bilateral mid and superior temporal lobe revealed a quadratic peak in mid-adolescence, with relatively stronger activation for self than mother in mid-adolescence. Prior studies showed a mid-adolescent peak in striatum (caudate) activation in response to winning for oneself (Braams *et al.*, 2014). Additionally, a prior study showed stronger right ventral striatum (caudate) activation for social trait evaluations from the perspective of a close other (best friend; Jankowski *et al.*, 2014). Striatum activation is also linked to processing self-relatedness (de Greck *et al.*, 2008), self-relevance (Enzi *et al.*, 2009) and intrinsic value (Zink *et al.*, 2003; Phan *et al.*, 2004). Therefore, the mid-adolescent peak in striatum for evaluating self *vs* mother might indicate that evaluating the self is relatively more self-relevant, salient or rewarding in mid-adolescence (Jankowski *et al.*, 2014). ACC activation (especially dorsal ACC) in the context of evaluating the self *vs* a close or public other has been interpreted as a top-down attentional process, where individuals consciously select traits that specifically fit one’s own personality, separate from others (Murray *et al.*, 2012). Our results suggest that mid-adolescents engage this process relatively more for self than mother, in line with stronger egocentrism and self-other differentiation at this age and the process of finding one’s own identity separate from one’s parents (Harter, 2012).

Additionally, there was a mid-adolescent peak for self *vs* mother in bilateral STG/MTG, regions encompassed in the superior temporal sulcus (STS; Allison *et al.*, 2000). The STS is considered part of the social brain network (Blakemore, 2008), which performs several functions including mentalizing (Redcay, 2008) and representing others’ emotional states (Peelen *et al.*, 2010; Zaki *et al.*, 2010). Possibly, mid-adolescents do not take their mothers’ emotional states into account when evaluating mother traits as much as early and late adolescents (right STS). Alternatively, mid-adolescents may take emotional states or opinions of others into account relatively more for self traits than do early and late adolescents (left STS; Harter, 2012).

Together, these results show that regions involved in self-reference, self-relevance and mentalizing are engaged relatively more for self than mother in mid-adolescents compared to early and late adolescents. Future longitudinal studies could investigate whether these neural and related behavioral mechanisms in the mother–adolescent relationship are related to adolescent egocentrism within individuals (Harter, 2012), providing more insight into the origins of this phenomenon.

### Strengths and limitations

The strength of this study was its relatively large fMRI sample size, broad focus on the whole range of adolescence and combination of neural, self-report and observation measures. This study, however, also had several limitations, which provide directions for future research. One limitation of the current study is the use of univariate data analyses. A more advanced approach would be the use of multivariate pattern analyses such as representational similarity analysis (Kriegeskorte *et al.*, 2008). This method would enable the construction of actual overlapping ‘patterns’ of neural activation for evaluating the self and evaluating a close other, which would give a more complete image of the neural mechanisms underlying self- and close-other processing compared to the univariate method. Therefore, we recommend that future studies on this topic be optimally designed for conducting multivariate analyses. Moreover, to better understand the relationship between behavioral and neural indicators of mother–adolescent relationships, future longitudinal studies could aim to investigate possible mediating effects of neural activation in the relationship between mother and adolescent behavior.

In addition, future research could also collect neural measures for mothers’ evaluations about their children. Although this study already used three types of measurements (self-report, observation and fMRI) neuroimaging measures from the mother can provide a more complete impression of the relationship. Moreover, relationships with fathers have different effects than mothers on adolescent’s psychological development (e.g. identity exploration and emotional stability), and this may also differ for boys and girls (Grotevant and Cooper, 1985; Laursen *et al.*, 1998; Hay and Ashman, 2003; De Goede *et al.*, 2009). A larger sample size would allow for the inclusion of fathers and for distinguishing between mother–daughter, mother–son, father–daughter and father–son relationships.

Finally, future research might focus on multiple forms of close relationships, for example relationships of adolescents with parents, siblings, close friends and classmates. This could shed light on potential shifts in closeness for different kinds of relationships. For example, it might be the case that adolescents have more negative social relationships with all close others in mid-adolescence, due to an excessive focus on the self (Harter, 2012).

## Conclusions

To conclude, this study showed that similarly strong mPFC activation for self and mother reflects closeness or warmth in the mother–adolescent relationship (Harris and Fiske, 2007; Krienen *et al.*, 2010; Raposo *et al.*, 2011). Additionally, this study revealed that the relative dip in relationship quality in mid-adolescence (Larson *et al.*, 1996; Laursen *et al.*, 1998; Shanahan *et al.*, 2007) co-occurs with relatively stronger neural activation for evaluating self *vs* mother traits in regions involved in self-reference, self-relevance and mentalizing (Denny *et al.*, 2012). These findings provide insight into the neural changes occurring in a time of social-cognitive changes and changes in mother–adolescent relationship closeness. Additionally, the current results put previous behavioral notions of greater self-consciousness or self-focus in mid-adolescence (Vartanian, 2000) in a social neuroscience perspective, indicating in concordance with previous studies that the enhanced self-focus seems to be reflected on the neural level as well (Somerville *et al.*, 2013).

## Supplementary Material

scan-18-338-File009_nsz023
