# Supplementary material for: Neural responses for evaluating self and mother traits in adolescence depend on mother–adolescent relationships
Source: Soc Cogn Affect Neurosci. 2019 Apr 4;14(5):481–92. doi: 10.1093/scan/nsz023 (PMC6570819; doi:10.1093/scan/nsz023)
Supplement: scan-18-338-File009_nsz023 [file scan-18-338-file009_nsz023.doc]

**Supplement**

**fMRI Data Acquisition and preprocessing**

MRI scans were collected using a Philips 3T MRI scanner, with a standard whole-head coil. Functional scans were acquired in three runs with T2*-weighted echo-planar imaging (EPI) sequence (TR = 2200 msec, TE = 30 msec, sequential acquisition, 37 slices of 2.75 mm, FOV = 220 x 220 x 111.65 mm). To account for T1 saturation, the first two volumes were discarded. For anatomical reference, a high-resolution 3D T1-FFE scan was obtained after the functional scans (TR = shortest msec, TE = 4.6 msec, 140 slices, voxel size = 0.875 mm, FOV = 224 x 178.5 x 168 mm). Sentences were projected on a screen behind the scanner and could be seen by the participant via a mirror attached to the head coil. Placing foam inserts inside the coil restricted head movement.

The data were analyzed using SPM8 (Wellcome Department of Cognitive Neurology, London). All functional scans were corrected for slice-timing acquisition and differences in rigid body movement. All structural and functional volumes were spatially normalized to T1 templates. The normalization algorithm used a 12-parameter affine transformation together with a nonlinear transformation involving cosine basis functions. The algorithm resampled the volumes to 3 mm cubic voxels. Templates were based on the MNI305 stereotaxic space (Cocosco et al., 1997). Functional volumes were spatially smoothed with a 6 mm FWHM isotropic Gaussian kernel.

**Supplementary results**

Adolescents whose mothers were more negative in their communication, showed relatively more bilateral middle occipital lobe activation for evaluating the Self > Mother, compared to adolescents whose mothers showed less negativity in their communication. Follow-up correlations within the ROIs extracted from this analysis for Self>Control and Mother>Control separately showed that the effect in left occipital lobe was driven by a decrease in activation for Mother>Control as mothers show more negativity in interaction with their adolescents (Self>Control *r*=.127, *p*=.224, Mother>Control *r*=-.217, *p*=.037), whereas the effect in right occipital lobe was driven by an increase in activation for Self>Control as mothers show more negativity in interaction with their adolescents (Self>Control *r*=.225, *p*=.030, Mother>Control *r*=-.114, *p*=.227). Follow-up t-tests in five groups showed that adolescents whose mother show little negativity in interaction engage right occipital lobe stronger (*p*=.025) and left occipital lobe marginally stronger (*p*=.069) for mother compared to self, whereas adolescents whose mothers show more negativity in interaction showed stronger bilateral occipital lobe activation for self compared to mother (right: *p*=.009; left: *p*=.001). Adolescents’ negativity in interaction, mothers’ and adolescents’ warmth in interaction did not result in significant brain activation in this contrast.

Suppl. Table 1
*Regions activated during the Self>Control and Mother>Control contrast*

|  | *Region* | *BA* | *Coordinates* | | | *Cluster Size* | *T* |
| --- | --- | --- | --- | --- | --- | --- | --- |
| *A. Self>Control  FDRc = 66* |  |  |  | | |  |  |
| Frontal/ | R Superior Medial Frontal (mPFC) | 10 | 6 | 62 | 13 | 1105 | 7.09 |
| Subcortical | L Anterior Cingulum | 32 | -6 | 44 | 1 |  | 6.62 |
|  | L Superior Medial Frontal | 10 | -9 | 56 | 7 |  | 6.40 |
|  | R Inferior Frontal | 44 | 57 | 11 | 22 | 167 | 6.31 |
|  | R Superior temporal pole | 44 | 54 | 5 | -2 |  | 3.95 |
|  | R. Heschl | 41 | 54 | -4 | 4 |  | 3.87 |
|  | L Mid Frontal | 10 | -27 | 47 | 31 | 157 | 5.52 |
|  | L Superior Frontal | 9 | -21 | 44 | 40 |  | 4.90 |
|  | L Mid Frontal | 10 | -39 | 44 | 19 |  | 3.27 |
|  | L Insula | 44 | -42 | 8 | 4 | 83 | 4.39 |
|  | L Suppl. Motor Area | 6 | -9 | 2 | 67 | 100 | 6.72 |
| Parietal cortex | R Supramarginal gyrus | 40 | 60 | -28 | 46 | 297 | 6.60 |
|  | R Inferior Parietal | 40 | 51 | -43 | 55 |  | 4.92 |
|  | R Inferior Parietal | 40 | 54 | -46 | 46 |  | 4.34 |
| Temporal cortex | L Superior Temporal | 41 | -48 | -16 | 4 | 66 | 4.10 |
|  | L Superior Temporal | 40 | -51 | -22 | 10 |  | 3.34 |
| *B. Mother>Control*  *FDRc = 92* | |  |  |  |  |  |  |
| Frontal/ | R Superior Medial Frontal (mPFC) | 10 | 6 | 59 | 13 | 432 | 6.61 |
| Subcortical | L Superior Medial Frontal | 10 | -9 | 56 | 7 |  | 5.43 |
|  | L Anterior Cingulum | 24 | -3 | 38 | 1 |  | 5.12 |
|  | R Inferior Frontal | 44 | 57 | 11 | 22 | 92 | 3.21 |
|  | R Inferior Frontal | 44 | 51 | 8 | 4 |  | 3.89 |
| Parietal cortex | R Supramarginal gyrus | 40 | 60 | -28 | 46 | 181 | 6.24 |
|  | R Inferior Parietal |  | 51 | -46 | 55 |  | 3.81 |
|  | R Supramarginal gyrus | 40 | 45 | -40 | 43 |  | 3.72 |

Names were based on the Automatic Anatomical Labeling (AAL) atlas.

Suppl. Table 2
*Regions activated during the (Self – Control) > (Mother - Control) and (Mother – Control) > (Self>Control) contrast*

|  | *Region* | *BA* | *Coordinates* | | | *Cluster Size* | *T* |
| --- | --- | --- | --- | --- | --- | --- | --- |
| *A. Self>Mother  FDRc = 29* |  |  |  | | |  |  |
| Frontal/ | **L Superior Frontal** |  | -15 | 35 | 37 | 151 | 4.84 |
| Subcortical | L Superior Frontal |  | -12 | 26 | 55 |  | 4.25 |
|  | L Superior Frontal |  | -27 | 47 | 28 |  | 4.21 |
|  | **L Supplementary Motor Area** |  | -12 | 11 | 64 | 34 | 4.81 |
|  | **L Precentral** |  | -33 | -1 | 43 | 154 | 4.83 |
|  | L Mid Frontal |  | -45 | 14 | 46 |  | 4.34 |
|  | L Mid Frontal |  | -27 | 17 | 37 |  | 3.73 |
|  | **R Mid Frontal** |  | 33 | 8 | 46 | 67 | 4.01 |
|  | R Mid Frontal |  | 39 | 20 | 43 |  | 3.81 |
|  | **R Supplementary Motor Area** |  | 12 | 14 | 61 | 29 | 3.80 |
|  | **R Caudate** |  | 21 | 2 | 22 | 33 | 3.75 |
|  | R Caudate |  | 27 | -4 | 25 |  | 3.74 |
|  | **L Mid Cingulum** |  | 0 | -22 | 40 | 57 | 4.16 |
|  | L Mid Cingulum |  | 0 | -28 | 34 |  | 3.90 |
| Parietal cortex | **L Postcentral** |  | -45 | -16 | 31 | 84 | 4.75 |
|  | **L Angular** |  | -45 | -64 | 46 | 93 | 4.09 |
|  | L Angular |  | -51 | -67 | 34 |  | 3.75 |
|  | L Inferior Parietal |  | -48 | -55 | 46 |  | 3.66 |
| Occipital cortex | L Mid Occipital |  | -30 | -97 | 1 | 89 | 5.37 |
| *B. Mother>Self*  *FDRc = 375* | |  |  |  |  |  |  |
| Occipital cortex | R Calcarine |  | 15 | -88 | 13 | 375 | 7.16 |
|  | R Lingual |  | 12 | -79 | -8 |  | 6.91 |

Names were based on the Automatic Anatomical Labeling (AAL) atlas.
